# Multiple cartilaginous exostoses in a Swiss Mountain dog causing thoracolumbar compressive myelopathy

**DOI:** 10.1186/s13028-019-0467-z

**Published:** 2019-06-25

**Authors:** Adriana Czerwik, Agnieszka Olszewska, Barbara Starzomska, Rafał Korta, Manfred Henrich, Marcin Wrzosek, Martin Jürgen Schmidt

**Affiliations:** 1 Department of Internal Medicine and Clinic of Horses, Dogs and Cats, Faculty of Veterinary Medicine, Wrocław University of Environmental and Life Sciences, pl. Grunwaldzki 47, 50-366 Wrocław, Poland; 2 Department of Veterinary Clinical Sciences, Small Animal Clinic, Justus-Liebig-University, Frankfurter Str. 108, 35392 Giessen, Germany; 3 Veterinary Clinic Kleintierzentrum Bruck, Sportplatz Str. 1b, 5671 Bruck an Der Grossglocknerstrasse, Austria; 4 Veterinary Clinic Arwet, ul. Sienkiewicz 25, 32-020 Wieliczka, Poland; 5 Department of Veterinary Pathology, Small Animal Clinic, Justus-Liebig-University, Frankfurter Str. 96, 35392 Giessen, Germany

**Keywords:** Computed tomography, Dog, Magnetic resonance imaging, Multiple cartilaginous exostoses, Surgery

## Abstract

**Background:**

Multiple cartilaginous exostoses are a rare, benign, proliferative condition of cartilage and bone. They can be asymptomatic, or they may cause pain, lameness, paresis and even paralysis, depending on their location and size. In cases of spinal cord or nerve root compression, surgery is the treatment of choice. Therefore, an advanced imaging diagnostic work-up is indicated. Due to the unclear pathophysiology and progression of this condition, it is difficult to predict its prognosis.

**Case presentation:**

A 9-month-old female Swiss Mountain dog was presented with a history of gait abnormalities, kyphosis and hypersensitivity consistent with a thoracolumbar myelopathy. Multiple calcified masses, most prominent at the Th7–Th9 level and the L2–L3 level, were observed. Magnetic resonance imaging of the thoracolumbar vertebral column revealed severe dorsal spinal cord compressions near the dorsal arch of the Th7–Th9 and L2–L3 vertebrae. Two of these masses were removed surgically. The successful removal of both masses was confirmed by postoperative computed tomography. The histopathological examination of the resected tissue revealed multiple cartilaginous exostoses. The first neurological and magnetic resonance follow up examination carried out 6 months postoperatively showed improvement of the clinical status. At that time, no mass regrowth was observed. The last follow up neurological examination carried out 15 months postoperatively showed gait improvement and resolution of pain.

**Conclusion:**

This is the first case report of multiple cartilaginous exostoses with a complete pre- and postoperative evaluation and a 15 month follow-up.

## Background

Multiple cartilaginous exostoses (MCE) is a rare, benign, proliferative condition of cartilage and bone described in humans, horses, dogs and cats [1, 2]. In the literature, this disorder is known as ostechondromatosis, osteocartilaginous exostoses, osteochondroma and diaphyseal aclasia [3]. The first clinical signs in affected dogs appear in or after the growth phase [4]. Although the pathophysiology is not completely understood, the disease is associated with abnormal migration of chondrocytes from the epiphyseal growth plates towards the bony cortex. Ossification of heterotopic cartilage cells causes subsequent irregular bone formation on bone surfaces [4–6]. These changes mainly involve tissues that undergo endochondral ossification, i.e., long bones, ribs and vertebrae (especially spinous processes, as well as the vertebral bodies and arches) [4, 5, 7]. Bones of an intramembranous origin (i.e., facial bones) are not affected. Recently, a de novo mutation in the *EXT2* gene was reported in a litter of American Staffordshire Terrier dogs with MCE. This gene is strongly associated with MCE in humans [8]. While the growth of most exostoses discontinues as the animal matures, some lesions may undergo neoplastic transformation as was observed in a group of adult dogs over 7 years old [7]. In cats, the disease was reported to be associated with a feline leukemia virus infection [9].

There are few reports concerning the diagnosis and treatment of cartilaginous exostoses in dogs. This case report describes the clinical and neurological presentation, imaging and histopathological diagnostic findings, surgical outcome and imaging findings after surgery in a dog with MCE.

## Case description

**Fig. 1:**
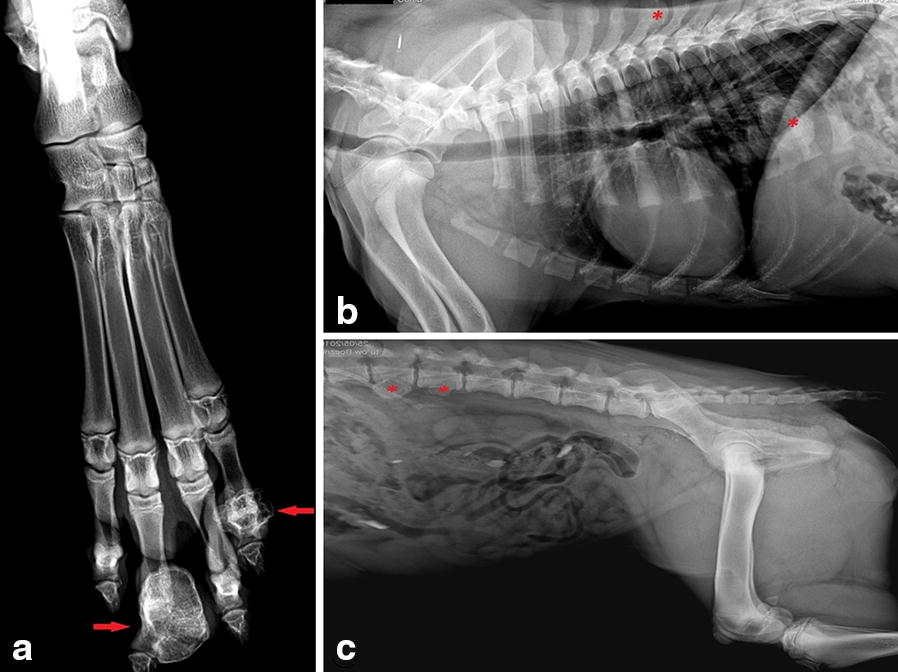
Radiographs of the right pelvic limb. **a** Showing two proliferative mass lesions within the II and IV phalanges (red arrows); **b** thoracic X-ray (lateral view) with two marked exostoses (*) at the level of the sixth thoracic spinous process and at the level of the ninth rib; **c** abdominal radiographs (lateral view) with marked (*) exostoses at the level of the second and third lumbar vertebral bodies

A 5-month old female Swiss Mountain dog presented to the local veterinary practice for clinical evaluation of gait abnormalities of the hind limbs. The orthopedic examination revealed lameness in the lower right hind limb and a bony thickening at the level of the metatarsophalangeal joint between the II and III digit phalanges, covered with normal skin. The complete blood count, serum chemical and electrolyte analyses were within normal limits, apart from elevated levels of calcium and phosphorus, which were attributed to the young age (5 month) of the animal (Table 1). The dog underwent a radiographic examination. Two well-defined lesions within the right II and IV phalanges of the right pelvic limb were found (Fig. 1a), in the shape of well circumscribed, mineralized new bone formation with a trabecular pattern. The dog underwent surgery to remove the lesion on the II phalange, and the excised tissue was sent for a histopathological examination. This revealed well-differentiated bone tissue, hyaline cartilage, and fibrous tissue in addition to hematopoietic tissue and connective tissue. Based on the histopathological findings, multiple cartilaginous exostoses (ostechondromatosis) were diagnosed. In the 1 month follow-up examination, the dog showed progressive ataxia of hind limb, a stiff gait and hyperesthesia in the thoracolumbar vertebral column. Radiographs of the hips and vertebral column exposed similar lesions to those in the pelvic limb. They were located in the distal part of the femur, on the tail and on the thoracic spinous processes of the lumbar vertebral bodies (Fig. 1b, c) and on the ribs (Fig. 1b).

**Table 1:** Reference values of phosphorus and calcium levels in immature and adult dogs compared with those of the 5-month old patient (Idexx Laboratory)

	Immature dog	Patient	Adult dog
Phosphorus concentrations (mmol/L)	1.29–3.1	3.1	0.97–1.94
Total calcium concentrations (mmol/L)	2.35–2.98	3.0	2.25–2.88

At the age of 8 months, the dog was presented to the Wrocław University of Environmental and Life Sciences at the Department of Internal Medicine with Clinic of Horses, Dogs and Cats for neurological assessment. The neurological examination revealed a wide-based stance and proprioceptive ataxia in the hind limbs, moderate proprioceptive deficits in the hind limbs, normal spinal reflexes and hypersensitivity of the thoracolumbar part of the vertebral column during palpation. The symptoms were localized to the Th3–L3 spinal cord segments.

**Fig. 2:**
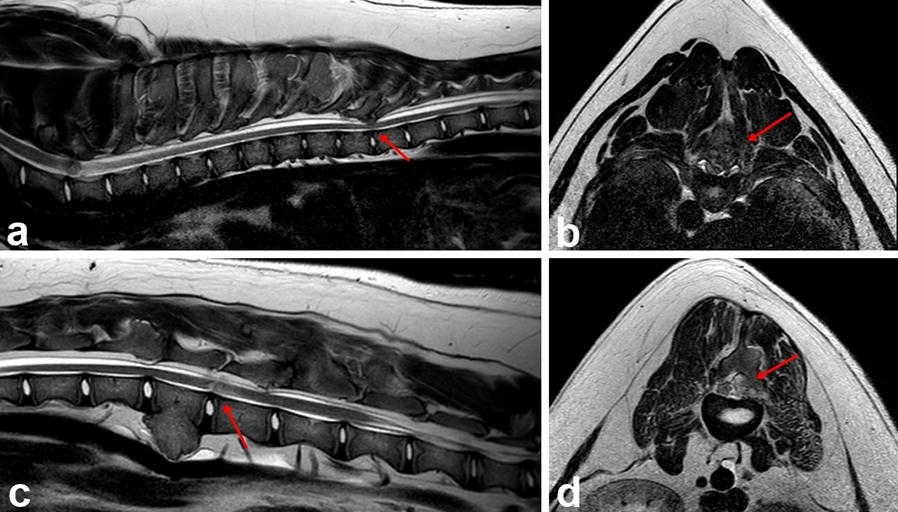
T2-weighted sagittal (**a**, **c**) and transverse (**b**, **d**) MR-images. **a** There is a deviation of the Th8–Th9 vertebral arch that protrudes ventrally into the vertebral canal. There is loss of visualization of the epidural fat and CSF column dorsally and hyperintense intramedullary signal (red arrow); **b** there is a large bone mass causing severe extradural dorsal right compression of the spinal cord at the level of the Th8–Th9 intervertebral space (red arrow); **c** visible hyperintense intramedullary signal between the L2–L3 intervertebral spaces (red arrow); **d** a large bone mass causing severe extradural right compression of the spinal cord at the level of L2–L3 intervertebral space (red arrow)

Magnetic resonance imaging (MRI) was performed using a high-field 1.5 Tesla MR scanner (Ingenia; Philips Medical Systems, 2014). The sequences included sagittal plane T1- and T2-weighted (T1W, T2W) images as well as dorsal and transverse plane T2W images. MRI changes involved various heterogenous, hyper- and hypointense lesions in the T2W sequence and hypo- and isointense lesions in the T1W sequence located on the vertebral body, arches and numerous spinous processes in the cervical, thoracic and lumbar regions causing deformation and various degrees of spinal cord compression. Moderate spinal cord compression was found on the left side at the level of the C5–C6 and C6–C7 intervertebral space. There was severe compression on the dorsal right side at the level of the caudal part of the Th8 vertebra and the Th8–Th9 intervertebral space (Fig. 2a, b), moderate compression on the right side at the level of the Th11 vertebra and dorsally at the level of the L1–L2 intervertebral space, severe compression on the right side of the L2–L3 intervertebral space (Fig. 2c, d) and moderate dorsal compression at the L4–L5 intervertebral space. Hyperintense intramedullary signal in the T2W sequence and isointense spinal cord signal in T1W sequence were detected in the compressed areas. There were severe, similar looking changes visible on the inner surface of the ribs and milder lesions were present on both sides of the lateral surface of the scapula and on the medial surface of the ribs. The medical imaging diagnosis included multifocal compressive myelopathy of the cervical, thoracic and lumbar segments of the spinal cord as a consequence of MCE, causing moderate to severe compression of the spinal cord at the C6–C7, Th8–Th9, Th11, L1–L2, L2–L3 and L4–L5 levels. Symptomatic medical management with prednisone (0.5 mg/kg twice daily), gradually tapered after 4 days, omeprazole (1 mg/kg once daily), gabapentin (10 mg/kg three times daily) and physiotherapy with restriction of movement was recommended. Decompressive surgery was recommended for spinal cord and nerve root entrapment.

**Fig. 3:**
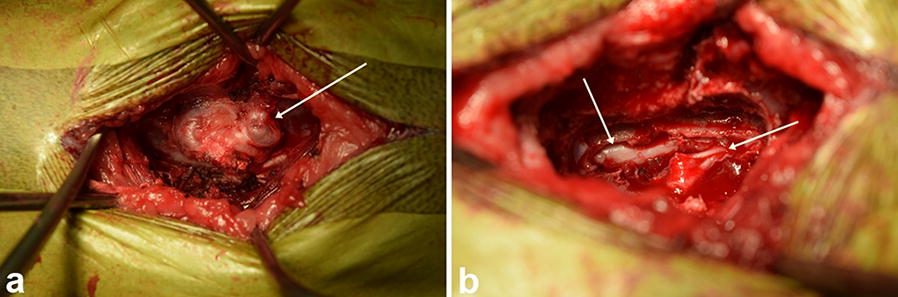
Intraoperative view of the vertebral column at the level of Th8–Th9 showing pathological exostoses located on the spinous processes (**a** yellow arrow). The spinal cord and nerve root are clearly visible after removal of the pathological exostoses (**b** yellow arrows)

**Fig. 4:**
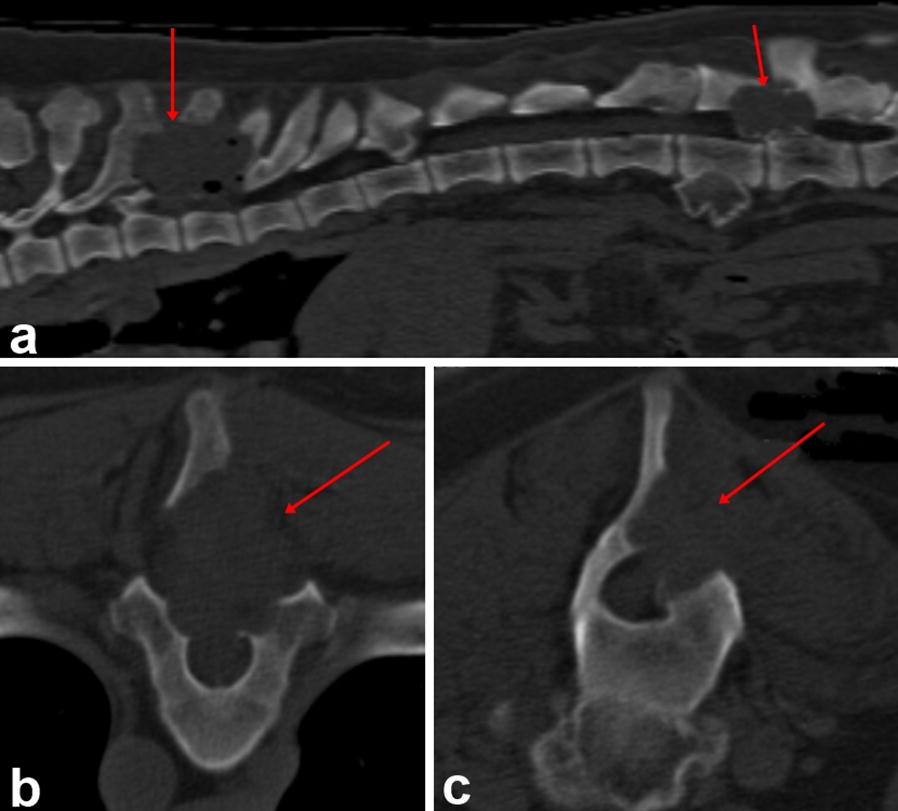
Sagittal (**a**) and transverse (**b**, **c**) CT images taken postoperatively showing the surgically removed bone and the osseous defects in the vertebral roof and spinous processes of the Th8, T9 and L2, L3 vertebrae. **b** Removal of the dorsal roof and part of the spinous processes of the vertebra at the level of Th8. **c** Excision of the vertebral roof and part of the spinous process of the vertebra at the level of L2

Three weeks after MRI examination, the dog underwent hemilaminectomy in the Department of Veterinary Clinical Sciences in the Small Animal Clinic of the Justus-Liebig-University Giessen in Germany. Due to the severity of lesions and neurolocalization in the thoracolumbar spinal cord, the surgical removal of the Th7–Th9 and L2–L3 lesions was performed via a dorsolateral approach (Fig. 3) with partial extension to the dorsal part of the vertebral lamina of Th7–Th9 and L2–L3 on the right side. Using an electrical burr, the bony protuberances were separated from the normal bone and removed en-bloc. Analgesic doses of morphine were administered locally (0.1 mg/kg). The excision was sent for a histopathological examination. The post-operative computed tomography (CT) was performed using a 16-slice Philips medical scanner (2006). It confirmed complete removal of the bone exostoses in the vertebral column at the level of Th7–Th9 and L2–L3 (Fig. 4). Intraoperative antibiotics (amoxicillin 25 mg/kg) were administered. The postoperative care involved intensive physiotherapy, administration of inflammatory (metamizole 50 mg/kg three times daily) and analgesic agents (fentanyl, ketamine, lidocaine). The dog was discharged after 2 days with gabapentin (10 mg/kg three times daily) maintained for 2 months and gradually tapered.

**Fig. 5:**
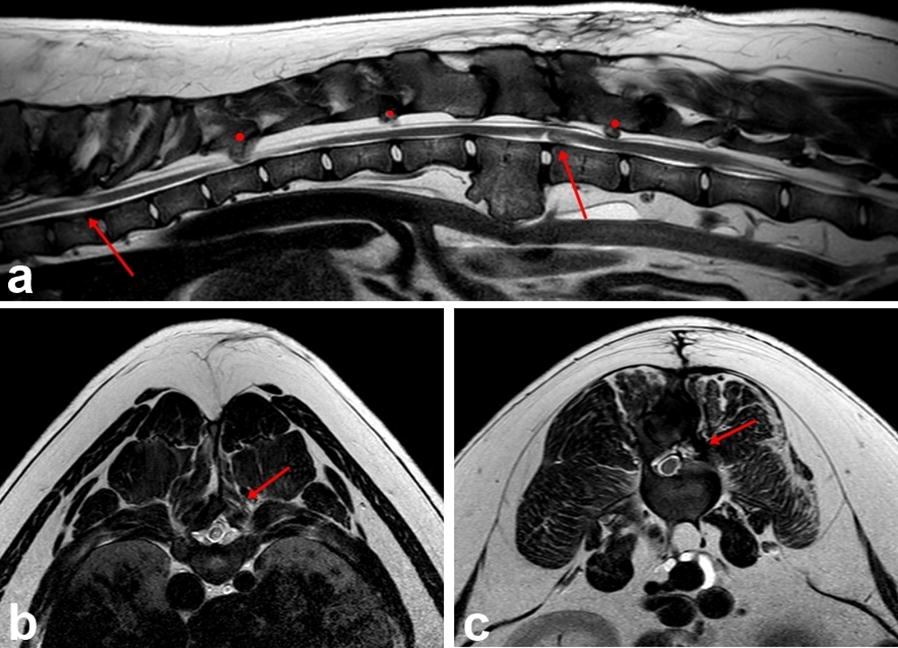
T2-weighted sagittal (**a**) and transverse (**b**, **c**) MR-images. **a** There is visible intramedullary signal between the Th8–Th9 and L2–L3 intervertebral space (red arrow). The remaining dorsal extradural compressions (red dot) are visible. They did not cause signs of pain during follow-up; **b** the large bone mass was removed at the level of the Th8–Th9 intervertebral space, resulting in spinal cord decompression. There is visible hyperintense intramedullary signal at this site (red arrow); **c** the large bone mass was removed at the level of the L2–L3 intervertebral space, showing lack of spinal cord compression (red arrow)

The first follow-up neurological and MRI examinations 7 months after the first MRI and 6 months post-surgery (when the dog was 15 months old) showed progressive, significant gait improvement with no signs of pain. The neurological investigation revealed a wide based—stance and mild proprioceptive ataxia in the hind limbs, normal proprioceptive positioning on all the limbs with normal spinal reflexes. Palpation of the vertebral column of the dog did not reveal any hyperesthesia or pain sings. The control MRI study revealed no further extension of the osteochondroma masses. A significant reduction in the spinal cord compression at the level of Th8–Th9 and L2–L3 intervertebral space was noted (Fig. 5). An intramedullary T2W hyperintense and T1W isointense signal was observed at both sites, indicating thoracolumbar myelopathy most likely secondary to previous compression of the spinal cord and subsequent spinal cord gliosis or edema. The compressions at the C6–C7 and L4–L5 level remained unchanged compared to the first MR examination. Regular, moderate physical activity and maintenance of a normal body weight was recommended. Based on a neurological examination performed 15 months post-surgery (when the dog was 2 years old), the dog did not show any further neurological deterioration.

## Discussion

Multiple cartilaginous exostoses causing solitary or multifocal and, in most cases, clinically silent lesions are a very rare disorder in young dogs. Occasionally, the development of the exostoses along the vertebral column may invade the vertebral canal and cause extradural spinal cord or nerve root compression. Neurological signs may appear in animals under 1 year of age due to discontinuation of further development of the normal growth plates [10]. However, neoplastic transformation may occur. MCE usually expands in the thoracolumbar vertebral column. The appearance and severity of the clinical signs depends on the localization of exostoses and their size [8]. Few reports of MCE with secondary myelopathy have been published in veterinary medicine, and its etiology has not been fully clarified. Some of the theories have been suggested [11, 12]. In dogs and humans, a hereditary basis is suspected in the case of multiple forms of exostoses [13–15]. Hence, breeding of affected dogs should be dissuaded.

Our patient revealed slightly elevated levels of calcium and phosphorus (Table 1), which were considered normal in relation to the dog’s growth. Decreased phosphorus and calcium levels were reported during maturation and immature animals should not be included in a reference for the adults [16, 17].

Clinical signs of MCE can vary depending on the localization and severity of the masses. In previously described cases, the clinical signs included progressive ataxia in four limbs with tetraparesis in three cases [13, 18, 19] indicating cervical neurolocalization and paraparesis in one case [20] indicating thoracolumbar neurolocalization. In our case, the dog presented with progressive ataxia in the hind limbs and moderate signs of pain in the thoracolumbar region. The presenting signs resulted from progressive development of the pathological masses and their secondary compression on the dura mater, nerve roots and/or spinal cord. A precise neurological examination is very important in cases with suspected MCE, because the masses can be multifocal, as in the presented case. The findings of the neurological evaluation and advanced diagnostic imaging enabled the localization of the pathological masses inflicting the largest damage to the physiology of the vertebral column.

A preliminary diagnosis of MCE is made based on imaging studies revealing characteristic vertebral column lesions. The bone masses are usually detectable on plain radiography, which can be used to evaluate the whole body in order to detect all pathological masses and can also rule out discospondylitis, which can cause very similar clinical signs to MCE in dogs. However, CT and MRI provide more detailed information about the involvement of the vertebral canal [9]. Both in human and veterinary medicine, MRI is considered a method of choice for central nervous system imaging, allowing an evaluation of the exact extent of the lesion as well as the location and the severity of spinal cord and nerve root compression. It is also very useful for surgical planning [9, 21, 22].

In the presented case, MCE was initially suspected based on the radiographs of the thoracolumbar region. Performing the MRI enabled a precise localization of the lesion prior to surgery. A hyperintense T2W, isointense T1W intramedullary signal was revealed, indicating spinal cord edema, gliosis or demyelination in the white matter and loss of nerve cells in grey matter [21, 23], which suggested spinal cord compression. Based on the neurological localization (Th3–L3) and the diagnostic imaging findings, the mass was excised.

Surgical excision and spinal cord decompression are crucial in animals with clinical evidence of spinal cord dysfunction. To date, there have been four reports of MCE in animals that affected the vertebral column and spinal cord. Three of those animals underwent successful surgical decompression procedures [13, 18, 19]. A post-operative vertebral fracture occurred in one case [20]. In the present case, we were able to follow the case until the dog was 15 months old and we performed several neurological examinations that revealed no deterioration of the neurological status and confirmed that a surgical intervention in the presented case was a good treatment option.

In this case, surgical treatment was necessary due to progressive signs of pain, gait abnormality and severe spinal cord compression in two regions, confirmed in MRI. This procedure prevented sudden decompensation and deterioration of the neurological status of the dog. Early surgical intervention may eliminate the evolution of clinical complications and eradicate a latent threat of malignancy in older dogs [8]. The nervous tissue is resistant to damaging effects of gradual compression, and severe compression is tolerated until sudden decompensation [13]. It has been suggested [19, 24] that surgical removal may be less challenging when the MCE are less developed, softer and with less vascularization. The lesions stop expanding as the animal matures, whereby the cancellous bone becomes harder and more vascular [8, 19, 24]. Prognosis is guarded, especially in young dogs with multiple vertebral involvement, where subclinical lesions may grow until the skeleton matures. For this reason, frequent monitoring of the neurological status along with MRI/CT imaging until the patient is mature is advised. In this case, the last neurological evaluation was performed more than 1 year after surgery and when the dog reached full maturity, indicating the effectiveness of the surgical treatment and no need for another surgery.

**Fig. 6:**
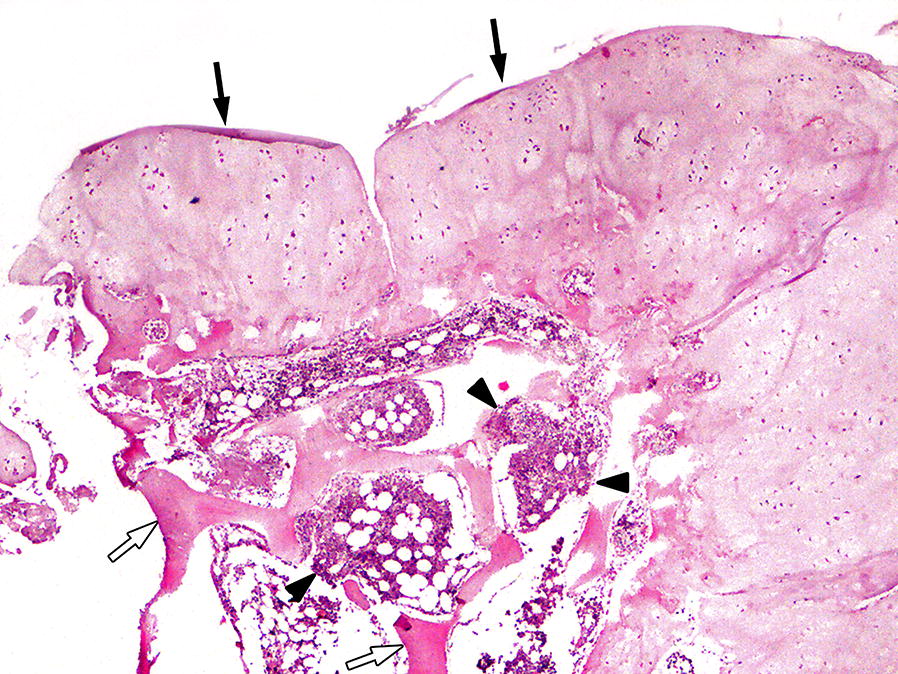
Photomicrograph of the excised tissue. The histopathological examination of the lesions revealed well differentiated bone tissue (white arrows), hyaline cartilage (black arrows), and fibrous cartilage in addition to hematopoietic tissue (black arrow heads) and connective tissue. No evidence of neoplasia or inflammation was found in the material samples tested. Based on histopathological findings, the diagnosis of multiple cartilaginous exostoses (ostechondromatosis) was made. Obj × 20, hematoxylin–eosin

It has been advocated that MCE should not be considered as neoplasms but rather as hamartomas [12]. However, definitive diagnosis is based on the histopathological examination [9], which allows to rule out the presence of neoplastic transformations of the masses. The histopathological appearance of MCE is typically described as caps of hyaline cartilage surrounding lamellar bone and intervening bone marrow [19, 25]. In the dog in this study, the histologic findings indicated Th7–Th9, L2–L3 vertebral body and phalange involvement, consistent with multiple cartilaginous exostoses (Fig. 6). No evidence of neoplasia or inflammation was found in the tested samples.

In summary, whole-body radiographs, CT and MRI should be the methods of choice for a presumptive diagnosis of MCE, as a methods that complement each other. Radiography, which is usually available in first-line veterinary practices, allows an initial diagnosis of MCE. CT is recommended when evaluating bony abnormalities. It provides detailed and specific information about the structure of bone tissue and the extent of bone lesions as it enables 3D image reconstruction, but it does not show the structure of the spinal cord. In addition, an MRI examination may help to determine the exact lesion localization for surgical planning and asses the parenchyma of the spinal cord. Decompressive surgery is advised in dogs with progressive neurological deterioration. Due to an unclear etiology of MCE and the expansion of the mass, further monitoring and frequent neurological follow-ups should be advised. This is the first case report presenting a long postoperative follow-up until full growth of the patient, confirming the effectiveness of the surgical treatment of MCE.

## Data Availability

All data generated or analysed during this study are included in this published article.

## Abbreviations

CT: computed tomography
MCE: multiple cartilaginous exostoses
MRI: magnetic resonance imaging
